# Psychological Distress of Metastatic Melanoma Patients during Treatment with Immune Checkpoint Inhibitors: Results of a Prospective Study

**DOI:** 10.3390/cancers13112642

**Published:** 2021-05-27

**Authors:** Lisa Wiens, Norbert Schäffeler, Thomas Eigentler, Claus Garbe, Andrea Forschner

**Affiliations:** 1 Department of Dermatology, University Hospital Tübingen, 72076 Tübingen, Germany; wiens_lisa@web.de (L.W.); thomas.eigentler@med.uni-tuebingen.de (T.E.); claus.garbe@med.uni-tuebingen.de (C.G.); 2 Department of Psychosomatic Medicine and Psychotherapy, University Hospital Tübingen, 72076 Tübingen, Germany; Norbert.Schaeffeler@med.uni-tuebingen.de

**Keywords:** metastatic melanoma, checkpoint inhibitors, immune therapy, psychooncology, distress, his, dt, problems

## Abstract

**Simple Summary:**

A large proportion of patients with metastatic melanoma suffer from psychological distress. Early identification of these patients is important to be able to offer them adequate support. This longitudinal study aimed to investigate the extent to which the psychological distress of patients with malignant melanoma might change during their first three months of treatment with immune checkpoint inhibitors. We found a high proportion of distressed patients in a cohort of 113 patients at the beginning of immunotherapy, which decreased during therapy. A binary logistic regression analysis provided additional factors indicating an increased risk of developing distress—female gender and occurrence of adverse events correlated significantly with distress values above the threshold. The strongest factor was patients’ self-assessment. When initiating immunotherapy, it is also important to consider the needs of patients and offer them psycho-oncological support.

**Abstract:**

Background: Immune checkpoint inhibitors (ICI) provide effective treatment options for advanced melanoma patients. However, they are associated with high rates of immune-related side effects. There are no data on the distress of melanoma patients during their ICI treatment. We, therefore, conducted a prospective longitudinal study to assess distress and the need for psycho-oncological support in these patients. Methods: Questionnaires were completed before initiation of ICI (T0), after 6–8 weeks (T1), and after 12–14 weeks (T2). We furthermore included the Hornheide Screening Instrument (HSI), distress thermometer (DT), and patients’ self-assessment. Binary logistic regression was performed to identify factors indicating a need for psychooncological support. Results: 36.3%/55.8% (HSI / DT) of the patients were above the threshold, indicating a need for psychooncological support at T0, and 7.8% of the patients reported practical problems. In contrast, at T2, the distress values had decreased to 29.0%/40.2% (HSI/DT), respectively. Female gender and occurrence of side effects significantly correlated to values above the threshold. The strongest factor was the patient’s self-assessment. Conclusion: With the beginning of ICI, psychooncological support should be offered. Furthermore, practical problems should be considered, e.g., transport to therapy. Female patients and patients with side effects should be given special attention, as well as the patient self-assessment.

## 1. Introduction

Melanoma is one of the most aggressive cancer types with an increasing incidence worldwide [1]. Receiving a diagnosis of melanoma is a deep cut in the lives of the person affected and can lead to numerous stress reactions [2]. Despite the high incidence of melanoma and an increasing number of new treatment options, only limited data is available on psychological distress in melanoma patients [3,4]. Some retrospective research indicated that patients on systemic therapy appear to be at lower risk for psychological distress, possibly due to the closer physician contact during therapy [5]. However, this was a retrospective survey that did not refer to the time of therapy initiation. 

The first-line treatment for patients suffering from melanoma is surgical excision of the primary tumor and, if present, of metastases [6]. For melanoma patients with stage III or IV and unresectable metastases, immune checkpoint inhibitors such as pembrolizumab or nivolumab (PD-1 Inhibitors) as monotherapy and nivolumab in combination with ipilimumab (CTLA-4 Inhibitor) are available and improved prognosis of metastasized melanoma patients considerably [7,8,9,10]. Pembrolizumab and nivolumab have also been approved in the adjuvant setting [6,11]. 

We aimed to investigate possible changes in psychological distress of melanoma patients during their first three months of treatment with immune checkpoint inhibitors.

The study was designed to provide a comprehensive overview of the patient cohort, including tumor-specific, social and psychological aspects, to better understand the individual psychooncological needs of the patients during their course of immune checkpoint inhibitor therapy.

## 2. Materials and Methods

We conducted a prospective, longitudinal study on melanoma patients from the Center for Dermatooncology at the University of Tuebingen.

Inclusion criteria were German-speaking women and men of full age who had been scheduled for treatment with immune checkpoint inhibitors (ICI) for metastatic melanoma (AJCC-Stage III/IV). Exclusion criteria were severe problems with the German language, severe psychological or neurological symptoms (e.g., psychosis, dementia), or lack of consent. The study was performed between December 2017 and February 2019. A total of 160 patients were informed about the study. Of these, 39 patients declined to participate. Thus, 121 patients decided to participate and provided written informed consent. Eight patients did not complete the first or second follow-up questionnaire, completed it only partially or too late, or decided against further participation in the study. The basis of the statistical evaluation was, therefore, 113 patients at T0. 

After detailed information about the possibility of participating in this study and the patient’s consent, the questionnaire was handed out before the start of ICI. The patient filled out the baseline questionnaire before the initiation of ICI, directly on site. It was directly transferred to the electronic Psychooncological Screening System (ePOS) (Supplementary File S1). 

The two follow-up questionnaires were conducted within 6–8 and 12–14 weeks after the initiation of ICI (Supplementary Files S2 and S3). Moreover, patients completed additional questionnaires at T1 and T2. In these questionnaires, the patients were asked to state whether they had had contact with a member of the psychooncological support team to that time point and whether the ICI therapy had been carried out as planned or not. Furthermore, they were asked to indicate whether side effects had occurred. During the survey, six patients died before completing the follow-up questionnaires. Nonetheless, they remained included. This is the reason for the statistical basis at time T1: 109 patients and T2: 107 patients.

Written consent was obtained from all patients included in this study, and ethical approval had been obtained from the local ethics committee of the Medical Faculty of the University of Tuebingen (file number 689/2017BO2).

### 2.1. Screening Instruments

#### 2.1.1. Distress Thermometer (DT)

The DT consists of a scale from 0–10. The patient is asked to indicate on the thermometer how much stress he/she has felt during the last week, including the day of survey. The value 0 indicates “not stressed at all”, the value 10 “extremely stressed” [12]. The DT is supplemented by an additional “problem list”, consisting of 36 items concerning family, emotional, spiritual, and physical aspects [13]. Following the recommendations of Mehnert et al. [13], we considered patients with DT values ≥5 as over-threshold, and thus, were in need of psychooncological support.

#### 2.1.2. Hornheide Screening Instrument (HSI)

The HSI represents the shortened form of the Hornheide Questionnaire (HF). The HSI is used to identify oncological patients in need of psychooncological support [14]. The seven items used in the HSI cover the following areas: 1—body condition, 2—mental condition, 3—disease-independent stress, 4—availability of a trusted person, 5—stress in the family due to the hospital stay, 6—the presence of inner peace, and 7—in what way the patient feels informed about the disease and treatment. For items 1, 2, and 7 the answer categories are 0 = rather good, 1 = medium and 2 = rather bad. The answer categories for items 3 and 5 are: 2 = Yes and 0 = No, for items 4 and 6: 0 = Yes and 2 = No. In computer-assisted screening, the analysis is carried out within seconds. The discriminant function on which the HSI is based identifies the patient as “in need of care” or “not in need of care”. The need for care is present with values >0.30 [15] using the discriminant function [16].

#### 2.1.3. Self-Assessment for the Need for Psychooncological Support

The subjective need for psychooncological support was assessed by the following question: “Do you currently need support in coping with the disease or psychooncological counselling?” [17].

### 2.2. Central Malignant Melanoma Registry

Tumor-specific data were obtained from the central registry for malignant melanoma. In this registry, information on the age and gender of the patients, date of the first diagnosis, metastasis, and further course of the disease are available. All patients included in this registry had provided written consent to this documentation.

### 2.3. Statistical Analyses

Demographic and clinical data were presented descriptively. The categorical variables were presented as absolute and relative frequencies, the continuous variables as mean values. A Mann–Whitney U test or Wilcoxon test was performed to analyze differences between patient groups (over-threshold values in DT present or absent). For small sample sizes, the exact Fischer test was used.

Furthermore, a binary logistic regression analysis was calculated to check for potential risk factors indicating the need for psychooncological support. Statistical analysis was performed using the statistical program for social sciences SPSS Version 25 (IBM, New York, NY, USA).

## 3. Results

One hundred and thirteen patients were included in the study, 54 women (47.8%) and 59 men (52.2%). The mean age of the women at the initial survey was 61.4 years (30–90 years, SD = 14.81), that of the men 62.4 years (30–89 years, SD = 13.80). 

**Figure 1:**
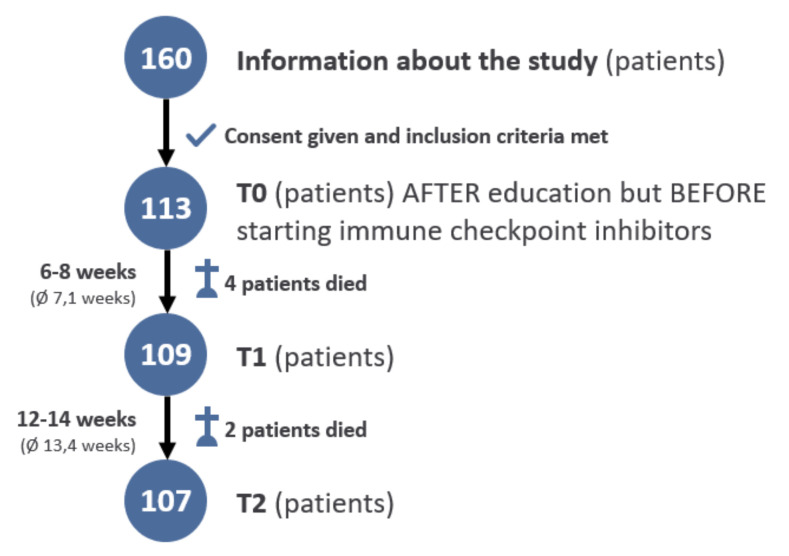
Flow chart.

Three patients were on daily antidepressants with citalopram or mirtazapine and one patient had a daily medication of lorazepam. Beyond that, none of the patients had a permanent medication with antidepressants or other psychopharmaceuticals. Of the 113 patients included at baseline (T0), 109 were alive at the first follow-up (T1) and 107 at T2 (Figure 1).

### 3.1. Clinical Data

**Figure 2:**
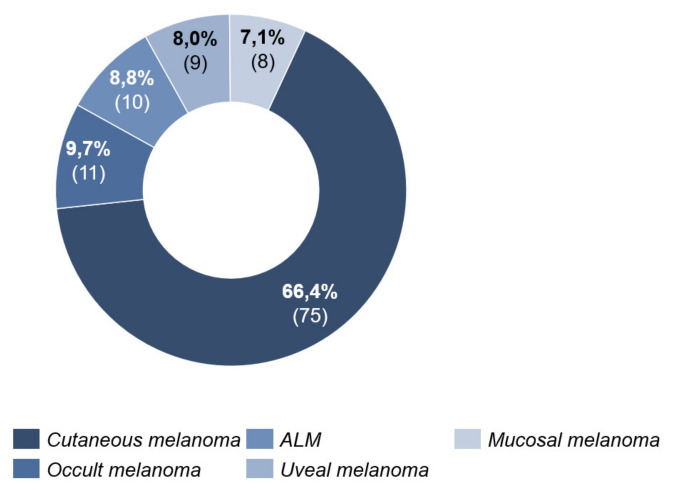
Melanoma type.

Cutaneous melanoma was the most common type (66.4%, *n* = 75) (Figure 2).

**Figure 3:**
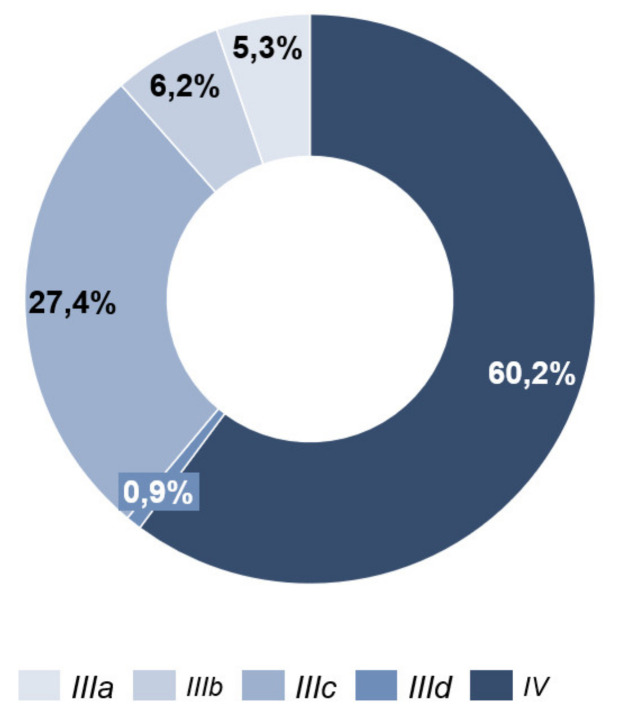
Tumor stage.

At the time of the initial survey (T0), 68 patients (60.2%) were classified as tumor stage IV and 45 patients (39.8%) as stage III (Figure 3).

**Figure 4:**
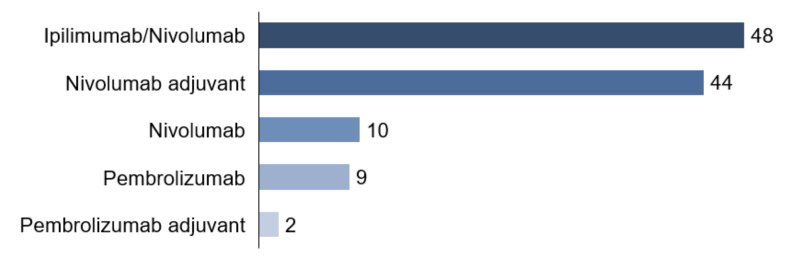
Therapy.

Combined systemic therapy with ipilimumab and nivolumab received 48 patients (42.5%). Ten patients (8.8%) started monotherapy with PD-1 antibody nivolumab, nine patients (8.0%) with PD-1 antibody pembrolizumab. Among the total cohort of 113 patients, 44 patients (38.9%) had nivolumab and two patients (1.8%) pembrolizumab in an adjuvant setting (Figure 4).

For evaluating the documented adverse events, patients were classified by treatment regime. On the one hand, patients with combined ICI (ipilimumab and nivolumab (*n* = 48)) and, on the other hand, patients who received a PD-1 antibody monotherapy with nivolumab or pembrolizumab (*n* = 65). 

**Figure 5:**
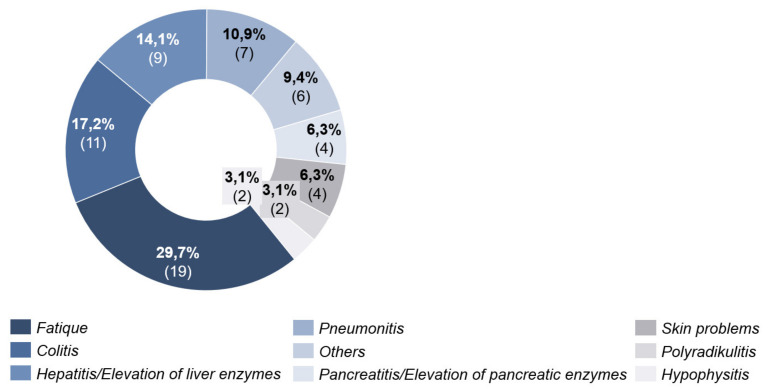
Side effects under combined immunotherapy.

In 65.2% (*n* = 30) of the patients with combined ICI, side effects occurred up to time T2. These appeared on average 33 days (4–63 days, SD = 18.14) after the start of systemic therapy. Most frequently (29.7%), fatigue was documented. The second most frequently documented side effect was colitis with 17.2% (Figure 5). 

**Figure 6:**
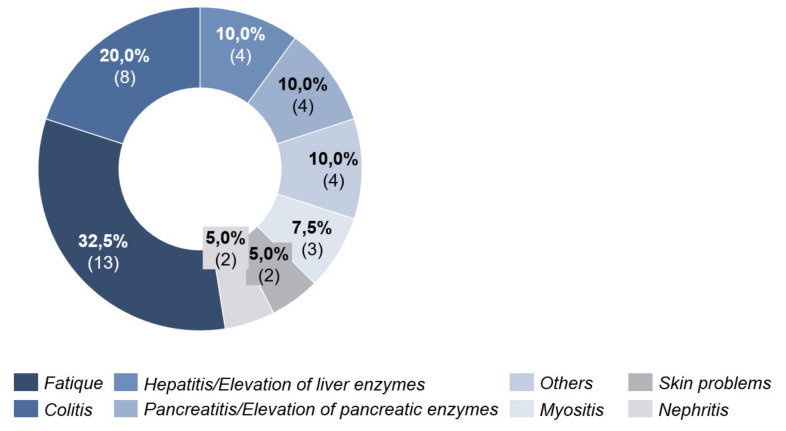
Side effects under PD-1 antibody therapy.

Under PD-1 antibody therapy, 27.0% (*n* = 17) of patients experienced side effects. These were seen after an average of 39 days (20–89 days, SD = 20.34) after initiation of ICI. Here, fatigue was also the most frequent cause with 32.5%. Colitis was in second place with 20.0% (Figure 6).

### 3.2. Psychological Distress

We found 55.8% of the patients were screened as significantly distressed by the Distress thermometer (cutoff ≥ 5). At T1, the percentage was 54.1%. At T2, the percentage dropped down to 40.2%. 

**Figure 7:**
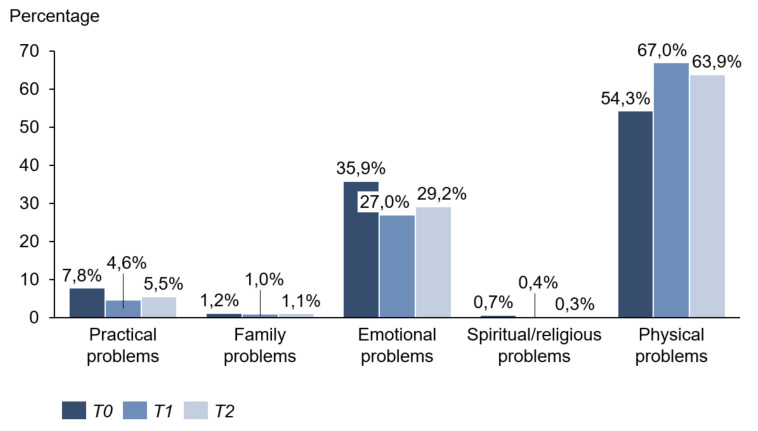
Problem areas.

Concerning the related problem list, physical problems were the priority at all measurement points. They reached their maximum value at time T1 (T0: 54.3%; T1: 67.0%; T2: 63.9%). Exhaustion, skin problems, sleep disorders, and pain were most frequently mentioned. Emotional problems came second to problem areas and were highest at the beginning of immunotherapy (T0: 35.9%; T1: 27.0%; T2: 29.2%). Above all, worries and fears occurred in half of the patients interviewed. Practical problems took third place. Family problems and religious issues were extremely rare (Figure 7 and Table 1).

Measured by HSI, 36.3% of patients showed over-threshold values (cutoff > 0.3) before starting therapy. At T1, the percentage was 36.7%. At time T2, the average exposure dropped to 29.0%.

Thirteen patients (11.5%) indicated a subjective need for psychooncological support at T0, nine patients (8.3%) at T1, and four patients (3.7%) at the last survey time T2. Within the survey period, twelve women and three men (13.8%; *n* = 15) had contact with a member of the psychooncological support team and had made an appointment for an interview. Of these 15 patients, seven had previously indicated a subjective need, another six patients showed over threshold values in HSI or DT, and one patient neither indicated a subjective need nor achieved values above the cutoff in the survey. At time T2, 21 patients (22.8%) who had not had contact with the psychooncological support team indicated that, in retrospective view, a conversation with the psychooncological team would probably have helped them to better cope with the disease.

The following table (Table 2) shows the individual values of the distress thermometer at all three survey times. In total, 63 patients marked a value of five or higher at T0 and were above the cutoff. At T1, there were 59 patients, and at T2, 43 patients with suprathreshold values.

### 3.3. Comparison of Groups with and without Over-Threshold Values in DT

Patients with (55.8%, *n* = 63) and without (44.2%, *n* = 50) over-threshold values in DT differed significantly in gender before the start of ICI. Women more often showed a value above the cutoff of ≥5 (Table 3). Significant differences were found in gender, tumor stage, type of therapy, and side effects at time T1 (Table 4). There were significant differences between the two groups in the occurrence of side effects and the outcome in staging at time T2 (Table 5). 

### 3.4. Indicators for the Need of Psychooncological Support According to HSI

A binary logistic regression was performed to check for the potential influence of patients’ subjective assessment and other objective factors on the need for psychooncological support. Since HSI is a widely used tool for assessing psychooncological support, we defined the HSI result as a response variable (HSI > 0.3: the need for care, HSI ≤ 0.3: no need for care).

#### 3.4.1. Parameters at the Beginning of Immunotherapy

The model included the following variables: positive subjective assessment, female gender, stage IV, and melanoma in the visible body parts. Other metric variables included age and duration of disease in months. The complete model, which included all parameters, was statistically significant, indicating that the model could distinguish between patients needing support and the patients not in need of psychooncological support. The model explained between 16.9% (Cox and Snell R-squared) and 23.2% (Nagelkerkes R-squared) of the variance and correctly classified 69.9% of cases. As shown in Table 6, 3 variables made a unique, statistically significant contribution to the model (positive subjective assessment, age at start of therapy, and female gender). According to HSI, the strongest predictor of psychooncological need was the variable “positive subjective assessment,” with an odds ratio of 5.76. This means that patients who reported a need for psychooncological support were more than five times more likely to achieve values above the threshold at HSI. Other significant parameters for values above the threshold in HSI indicating a need for psychooncological support were female gender and higher age. Increasing age increased the percentage of patients needing psychooncological support (Table 6).

#### 3.4.2. Parameters during Immunotherapy

The following variables were included in the model: positive subjective need, female gender, stage IV, side effects, and the type of therapy. The metric variable included age. The overall model was statistically significant, indicating that the model could distinguish between patients requiring care and those not requiring care. The model explained between 20.0% (Cox and Snell R-squared) and 27.3% (Nagelkerkes R-squared) of the variance and correctly classified 76.1% of cases. As shown in Table 7, 3 variables also made a statistically significant contribution to the model (positive subjective assessment, age at start of therapy, and the occurrence of side effects). The strongest predictor of psychooncological need after HSI was also the variable “positive subjective assessment” with an odds ratio of 8.21 (Table 7).

## 4. Discussion

In this prospective study on distress in metastatic melanoma patients during ICI, we identified more than 50% of distressed patients at baseline before initiating ICI. This percentage is slightly higher than that of other studies on the psycho-oncological burden of melanoma patients [2,5,18]. It can certainly be explained by the fact that our cohort included only advanced melanoma patients who were to receive systemic ICI. During ICI, the proportion of patients with values above the threshold decreased, whereas the physical problems increased to a clear peak at time T1.

It could be assumed that uncertainties about the detailed course and tolerability at the beginning of ICI (T0) led to a higher percentage of distressed patients.

When comparing the patient groups with and without DT values above the threshold, we found that female patients more often suffered distress than male patients at times T0 and T1. Consistent with our results, others have shown that women are distressed to a higher percentage than men during their disease [19,20,21]. One explanation for the remarkably higher percentage of female patients with DT values above the threshold could be that women might be more concerned about their families and children. Additionally, it has been shown that women are more restricted in their daily activities during their illness than men, which could explain an increased percentage of distressed patients [22].

Furthermore, patients with a higher AJCC stage (stage IV compared to stage III), combined immunotherapy (compared to PD-1 antibodies as monotherapy) and patients who developed side effects compared to those without, showed more distress at T1. We found a significant correlation between the type of immunotherapy and the occurrence of side effects. Thus, significantly more patients suffered from side effects when receiving combined immunotherapy. When comparing combined immunotherapy with monotherapy, it is noticeable that immune-related side effects occurred in 63% of patients with combined immunotherapy and 32% of patients with PD-1 antibody monotherapy. Similarly, several studies revealed that severe or life-threatening adverse events (CTCAE grade 3–4) occur in 17–21% with PD-1 antibody monotherapy and 59% with combined immunotherapy [23,24]. 

The immune-related side effects suffered by our patients occurred on average after 38 days. This timing of onset fits well with what has been reported in other studies, and it also correlates very well with the time-point of the second survey (T1). This certainly explains the peak of physical problems at time T1. In particular, colitis, and pneumonitis may entail prolonged adverse event management under inpatient conditions [25].

Interestingly, the proportion of patients indicating emotional problems decreases at time T1. In particular, the items “anxiety” and “depression” dropped at time T1 and rose again slightly at time T2, perhaps due to the approaching moment of staging.

One explanation could be that the patients focused on their physical, immune-related problems at T1 so that all other items of the problem list lost their importance beside it. Another explanation could be that patients associated and, to some extent, equated the occurrence of immune-mediated side effects with a good response to therapy.

In both combined immunotherapy and PD1-AK monotherapy, “fatigue” has been documented as the most common side effect. The same in other countries, regardless of the type of cancer, fatigue was the most often mentioned physical problem in cancer patients [26,27]. Nonetheless, fatigue seems to be still underdiagnosed, and support for these patients has not been adequately ensured [28]. Fatigue, in particular, is often stated by cancer patients as the main cause of impaired quality of life and increased distress [29].

The proportion of patients with progressive disease in staging showed an increase in DT at T2. In contrast, patients who showed a decrease in the tumor or metastases or who did not experience worsening the situation were significantly less distressed. This highlights the importance of empathic communication of the staging results in a face-to-face conversation and, if needed, prompt psycho-oncological support, which will primarily be the case with progressive findings.

Some factors can help to identify affected patients and thus to provide psychooncological support. The strongest predictor for patients above threshold by HSI, immediately before starting the systemic therapy (T0) and during its course, was the variable “subjective need.” For melanoma patients, who consider themselves to need psychooncological support, it can be assumed with high probability that they also have a psychometrically determined need, measured by HSI. In this context, a subjective need is of enormous importance and should be considered an independent, most probably even the simplest and most intuitive screening tool. This is also recommended in the guideline in the S3 guideline (“Psychooncological Diagnosis, Counseling and Treatment of Adult Cancer Patients”). Patients should be asked for their personal estimation on the need for psychooncological support [12]. However, this question alone should not replace the screenings as they contain several questions helping the patient be self-reflective. Likely, some patients would not have noticed that they had not been feeling well in the last few days, or they might have realized that they do NOT have anyone to talk to about their difficult situation. The screenings can help to induce a deeper self-reflection.

Therefore, at the beginning and during therapy, considering the patients’ subjective perception, psychooncological support should be offered. In this way, psychological comorbidities, such as anxiety or depression might be assessed early on. In this way moreover, compliance could probably be ensured. DiMatteo and colleagues found that patients with comorbid depression were three times more likely not to follow treatment recommendations or even discontinue therapy [30]. Through the availability of psychooncological support, patient care could be ensured, which could be lifesaving for cancer patients.

However, in contrast to the high proportion of patients with over threshold values in HSI and DT, only 11.5% indicated a subjective need for psychooncological support at the beginning of immunotherapy. This subjective need decreased even further during the treatment (T1: 8.3%; T2: 3.7%).

Possible reasons for patients not considering themselves to need support despite values above the threshold could be an insufficient knowledge about what the psychooncological support looks like in detail, a kind of fear of stigmatization, inability to cope with the disease and the resulting misjudgment, or other support they already receive [31,32].

The individual ability of the patients to cope with stress and use resources and possible intrinsic predispositions to feel anxious or worried may have biased the results of our study. Intrapsychic personality characteristics were not assessed. A follow-up study could focus on these possibly influencing factors in combination with further personality characteristics. 

During the survey period, more women than men made use of psychooncological support. This is in line with the results of the study by Zwaan et al., in which significantly more women than men had contact with the psychooncological service [33]. This gender difference is probably due to the traditional distribution of roles. For men, it might still be an obstacle to show weakness and ask for help. Based on these findings, patients should not only be relied on to seek psychooncological support themselves. They should be actively informed about possible supports and offers of help. Special care should be taken to reduce possible prejudices against “psychooncology” and to encourage men to seek external help.

## 5. Conclusions

Melanoma patients who are to be treated by ICI should be screened by DT or HSI for psychological distress, complemented by patients′ self-assessment. In our study, the percentage of values above the threshold decreased during ICI. At the beginning of ICI therapy, practical problems should be given special consideration, e.g., transport to therapy. It should be considered that there are special situations that might require additional support. Patients with immune-related adverse events had more often physical problems, and patients suffering progressive disease were significantly more often above-threshold, thus in need of psychooncological support.

A problem list, as in DT, can rapidly help the treating physician get an overview of the patient′s current problems. For the future, studies would be desirable to investigate the extent to which psycho-oncological support can reduce the proportion of over-threshold patients.

## Figures and Tables

**Table 1:** Problem list at T0, T1, T2.

Problem List	T0 (*n* = 113)	T1 (*n* = 109)	T2 (*n* = 107)
Practical problems			
Housing	4.4% (*n* = 5)	3.7% (*n* = 4)	3.7% (*n* = 4)
Insurance	3.5% (*n* = 4)	3.7% (*n* = 4)	5.6% (*n* = 6)
Work/school	13.3% (*n* = 15)	10.1% (*n* = 11)	13.1% (*n* = 14)
Transportation	16.8% (*n* = 19)	11.9% (*n* = 13)	10.3% (*n* = 11)
Childcare	1.8% (*n* = 2)	0.0% (*n* = 0)	0.0% (*n* = 0)
Family problems			
Dealing with partner	6.2% (*n* = 7)	4.6% (*n* = 5)	4.7% (*n* = 5)
Dealing with children	0.0% (*n* = 0)	1.8% (*n* = 2)	0.9% (*n* = 1)
Emotional problems			
Worry	51.3% (*n* = 58)	56.9% (*n* = 62)	62.6% (*n* = 67)
Fears	49.6% (*n* = 56)	47.7% (*n* = 52)	48.6% (*n* = 52)
Sadness	24.8% (*n* = 28)	23.9% (*n* = 26)	21.5% (*n* = 23)
Depression	10.6% (*n* = 12)	8.3% (*n* = 9)	9.3% (*n* = 10)
Nervousness	29.2% (*n* = 33)	19.3% (*n* = 21)	17.8% (*n* = 19)
Loss of interest in usual activities	14.2% (*n* = 16)	12.8% (*n* = 14)	5.6% (*n* = 6)
Spiritual/religious issues			
In relation to God	3.5% (*n* = 4)	2.8% (*n* = 3)	1.9% (*n* = 2)
Loss of faith	0.0% (*n* = 0)	0.0% (*n* = 0)	0.0% (*n* = 0)
Physical problems			
Pain	24.8% (*n* = 28)	40.4% (*n* = 44)	36.4% (*n* = 39)
Nausea	12.4% (*n* = 14)	22.9% (*n* = 25)	16.8% (*n* = 18)
Fatigue	40.7% (*n* = 46)	62.4% (*n* = 68)	64.5% (*n* = 69)
Sleep	31.9% (*n* = 36)	42.2% (*n* = 46)	43.9% (*n* = 47)
Getting around	25.7% (*n* = 29)	30.3% (*n* = 33)	20.6% (*n* = 22)
Bathing. dressing	3.5% (*n* = 4)	5.5% (*n* = 6)	4.7% (*n* = 5)
Appearance	3.5% (*n* = 4)	9.2% (*n* = 10)	4.7% (*n* = 5)
Breathing	11.5% (*n* = 13)	16.5% (*n* = 18)	10.3% (*n* = 11)
Mouth sores	1.8% (*n* = 2)	7.3% (*n* = 8)	1.9% (*n* = 2)
Eating	10.6% (*n* = 12)	13.8% (*n* = 15)	11.2% (*n* = 12)
Indigestion	12.4% (*n* = 14)	31.2% (*n* = 34)	33.6% (*n* = 36)
Constipation	9.7% (*n* = 11)	11.0% (*n* = 12)	5.6% (*n* = 6)
Diarrhea	9.7% (*n* = 11)	17.4% (*n* = 19)	12.1% (*n* = 13)
Changes in urination	9.7% (*n* = 11)	3.7% (*n* = 4)	3.7% (*n* = 4)
Fevers	0.0% (*n* = 0)	8.3% (*n* = 9)	6.5% (*n* = 7)
Skin dry/itchy	21.2% (*n* = 24)	45.0% (*n* = 49)	43.9% (*n* = 47)
Nose dry/congested	3.5% (*n* = 4)	11.0% (*n* = 12)	12.1% (*n* = 13)
Tingling in hands/feet	13.3% (*n* = 15)	11.9% (*n* = 13)	15.0% (*n* = 16)
Feeling swollen	7.1% (*n* = 8)	12.8% (*n* = 14)	12.1% (*n* = 13)
Memory/concentration	15.0% (*n* = 17)	15.6% (*n* = 17)	6.5% (*n* = 7)
Sexual	7.1% (*n* = 8)	5.5% (*n* = 6)	4.7% (*n* = 5)

**Table 2:** Distress thermometer at T0, T1, and T2.

DT Values	T0 (*n* = 113)	T1 (*n* = 109)	T2 (*n* = 107)
Value 0	2.7% (*n* = 3)	1.8% (*n* = 2)	1.9% (*n* = 2)
Value 1	6.2% (*n* = 7)	5.5% (*n* = 6)	10.3% (*n* = 11)
Value 2	8.8% (*n* = 10)	11.0% (*n* = 12)	15.0% (*n* = 16)
Value 3	13.3% (*n* = 15)	12.8% (*n* = 14)	14.0% (*n* = 15)
Value 4	13.3% (*n* = 15)	14.7% (*n* = 16)	18.7% (*n* = 20)
Value 5	17.7% (*n* = 20)	16.5% (*n* = 18)	20.6% (*n* = 22)
Value 6	15.9% (*n* = 18)	19.3% (*n* = 21)	13.1% (*n* = 14)
Value 7	9.7% (*n* = 11)	7.3% (*n* = 8)	3.7% (*n* = 4)
Value 8	4.4% (*n* = 5)	9.2% (*n* = 10)	2.8% (*n* = 3)
Value 9	4.4% (*n* = 5)	1.8% (*n* = 2)	0.0% (*n* = 0)
Value 10	3.5% (*n* = 4)	0.0% (*n* = 0)	0.0% (*n* = 0)

**Table 3:** Comparison of the groups at T0.

Characteristic	Overall *n* = 113 (100%)	DT < 5 *n* = 50 (44.2%)	DT ≥ 5 *n* = 63 (55.8%)	*p*-Value
Demographic characteristics				
Gender (%)				***p* = 0.041 ^C^** **V** = **0.210 ^1^**
Female	*n* = 54 (100%)	*n* = 18 (33.3%)	*n* = 36 (66.7%)	
Male	*n* = 59 (100%)	*n* = 32 (54.2%)	*n* = 27 (45.8%)	
Age (in Years)	61.9 (SD = 14.24)	61.6 (SD = 12.64)	62.2 (SD = 15.48)	*p* = 0.840 ^M^
Clinical characteristics				
Disease duration (in months)	38.2 (SD = 54.7)	35.8 (SD = 40.05)	40.1 (SD = 64.29)	*p* = 0.833 ^M^
AJCC-Stage				*p* = 0.317 ^C^
III	*n* = 45 (100%)	*n* = 23 (51.1%)	*n* = 22 (48.9%)	
IV	*n* = 68 (100%)	*n* = 27 (39.7%)	*n* = 41 (60.3%)	
Histological subtype				*p* = 0.415 ^C^
Cutaneous melanomas	*n* = 75 (100%)	*n* = 35 (46.7%)	*n* = 40 (53.3%)	
ALM	*n* = 10 (100%)	*n* = 2 (20%)	*n* = 8 (80%)	
Mucous membrane	*n* = 8 (100%)	*n* = 5 (62.5%)	*n* = 3 (37.5%)	
Uveal melanoma	*n* = 9 (100%)	*n* = 3 (33.3%)	*n* = 6 (66.7%)	
Occult melanoma	*n* = 11 (100%)	*n* = 5 (45.5%)	*n* = 6 (54.5%)	
Localisation				*p* = 0.192 ^C^
Head/neck	*n* = 21 (100%)	*n* = 8 (38.1%)	*n* = 13 (61.9%)	
Trunk	*n* = 31 (100%)	*n* = 18 (58.1%)	*n* = 13 (41.9%)	
Upper extremity	*n* = 10 (100%)	*n* = 5 (50%)	*n* = 5 (50%)	
Lower extremity	*n* = 32 (100%)	*n* = 9 (28.1%)	*n* = 23 (71.9%)	
Mucosa	*n* = 8 (100%)	*n* = 5 (62.5%)	*n* = 3 (37.5%)	
Occult melanoma	*n* = 11 (100%)	*n* = 5 (45.5%)	*n* = 6 (54.5%)	
Therapy				*p* = 0.294 ^C^
Combined immunotherapy	*n* = 48 (100%)	*n* = 18 (37.5%)	*n* = 30 (62.5%)	
PD-1-antibody monotherapy	*n* = 65 (100%)	*n* = 32 (49.2%)	*n* = 33 (50.8%)	

^C^ = Chi-Quadrat-Test. ^M^ = Mann-Whitney U Test. ^1^ V = Cramers V (Effect power: 0.10 small, 0.30 medium, 0.50 large).

**Table 4:** Comparison of the groups at T1.

Characteristic	Overall *n* = 109 (100%)	DT < 5 *n* = 50 (45.9%)	DT ≥ 5 *n* = 59 (54.1%)	*p*-Value
Demographic characteristics				
Gender (%)				***p*** = **0.041 ^C^** **V** = **0.210 ^1^**
Female	*n* = 50 (100%)	*n* = 16 (32.0%)	*n* = 34 (68.0%)	
Male	*n* = 59 (100%)	*n* = 34 (57.6%)	*n* = 25 (42.4%)	
Age (in Years)	62.45 (SD = 14.10)	61.3 (SD = 12.81)	63.4 (SD = 15.12)	*p* = 0.314 ^M^
Clinical characteristics				
AJCC-Stage				***p* = 0.004 ^C^** **V = 0.293 ^1^**
III	*n* = 44 (100%)	*n* = 28 (63.6%)	*n* = 16 (36.4%)	
IV	*n* = 65 (100%)	*n* = 22 (33.8%)	*n* = 43 (66.2%)	
Therapy				***p* = 0.003 ^C^** **V = 0.302 ^1^**
Combined immunotherapy	*n* = 46 (100%)	*n* = 13 (28.3%)	*n* = 33 (71.7%)	
PD-1-antibody monotherapy	*n* = 63 (100%)	*n* = 37 (58.7%)	*n* = 26 (41.3%)	
Side effects				***p* = 0.005 ^C^** **V = 0.287 ^1^**
No	*n* = 71 (100%)	*n* = 40 (56.3%)	*n* = 31 (43.7%)	
≥1	*n* = 38 (100%)	*n* = 10 (26.3%)	*n* = 28 (73.7%)	
Staging				*p* = 0.270 ^C^
No	*n* = 83 (100%)	*n* = 35 (42.2%)	*n* = 48 (57.8%)	
Stable Disease	*n* = 19 (100%)	*n* = 12 (63.2%)	*n* = 7 (36.8%)	
Progress	*n* = 7 (100%)	*n* = 3 (42.9%)	*n* = 4 (57.1%)	

^C^ = Chi-Quadrat-Test. ^M^ = Mann-Whitney U Test. ^1^ V = Cramers V (Effect power: 0.10 small, 0.30 medium, 0.50 large).

**Table 5:** Comparison of the groups at T2.

Characteristic	Overall *n* = 107 (100%)	DT < 5 *n* = 64 (59.8%)	DT ≥ 5 *n* = 43 (40.2%)	*p*-Value
Demographic characteristics				
Gender (%)				*p* = 0.057 ^C^
Female	*n* = 49 (%)	*n* = 24 (49.0%)	*n* = 25 (51.0%)	
Male	*n* = 58 (%)	*n* = 40 (69.0%)	*n* = 18 (31.0%)	
Age (in Years)	62.2 (SD = 14.08)	61.3 (SD = 14.15)	63.5 (SD = 14.02)	*p* = 0.429 ^M^
Clinical characteristics				
AJCC-Stage				*p* = 0.382 ^C^
III	*n* = 44 (100%)	*n* = 29 (65.9%)	*n* = 15 (34.1%)	
IV	*n* = 63 (100%)	*n* = 35 (55.6%)	*n* = 28 (44.4%)	
Therapy				*p* = 0.078 ^C^
Combined immunotherapy	*n* = 45 (100%)	*n* = 22 (48.9%)	*n* = 23 (51.1%)	
PD-1-antibody monotherapy	*n* = 62 (100%)	*n* = 42 (67.7%)	*n* = 20 (32.3%)	
Side effects				***p* = 0.000 ^C^** **V = 0.428 ^1^**
No	*n* = 83 (100%)	*n* = 59 (71.1%)	*n* = 24 (28.9%)	
≥1	*n* = 24 (100%)	*n* = 5 (20.8%)	*n* = 19 (79.2%)	
Staging				***p* = 0.001 ^C^** **V = 0.349 ^1^**
No	*n* = 88 (100%)	*n* = 54 (61.4%)	*n* = 34 (38.6%)	
Stable Disease	*n* = 9 (100%)	*n* = 8 (88.9%)	*n* = 1 (11.1%)	
Progress	*n* = 10 (100%)	*n* = 2 (20.0%)	*n* = 8 (80.0%)	

^C^ = Chi-Quadrat-Test. ^M^ = Mann-Whitney U Test. ^1^ V = Cramers V (Effect power: 0.10 small, 0.30 medium, 0.50 large.).

**Table 6:** Odds ratio for the need for psychooncological support according to the HSI.

Characteristic	Odds Ratio	Standard Error	*p*-Value	95% CI	Odds Ratio
Positiv subjective need	**5.76**	**0.76**	**0.020**	**1.31**	**25.36**
Female Gender	**2.93**	**0.47**	**0.022**	**1.17**	**7.33**
Higher Age before start therapy	**1.04**	**0.02**	**0.014**	**1.01**	**1.08**
AJCC-Stage IV	1.16	0.47	0.747	0.46	2.92
Longer duration of illness in month	1.00	0.00	0.678	0.99	1.01
Melanoma on visible parts of the body	0.45	0.60	0.179	0.14	1.45

The significant results are displayed first and marked in bold; sorted by odds ratio, starting with the highest value.

**Table 7:** Odds ratio for the need for psychooncological support according to the HSI during immunotherapy.

Characteristic	Odds Ratio	Standard Error	*p*-Value	95% CI	Odds Ratio
Positiv subjective need	**8.21**	**0.91**	**0.021**	**1.37**	**49.27**
Occurrence of side effects	**3.00**	**0.47**	**0.019**	**1.20**	**7.48**
Higher Age before start therapy	**1.04**	**0.02**	**0.037**	**1.00**	**1.08**
Combined immunotherapy	1.49	0.69	0.566	0.39	05.72
AJCC-Stage IV	1.01	0.70	0.985	0.26	4.02
Female gender	0.93	0.48	0.875	0.36	2.39
Staging	0.56	0.61	0.339	0.17	1.84

The significant results are displayed first and marked in bold, sorted by odds ratio, starting with the highest value.

## Data Availability

The datasets used and/or analyzed during the current study are available from the corresponding author on reasonable request. The data are not publicly available due to patient confidentiality.

## Supplementary Materials

The following are available online at https://www.mdpi.com/article/10.3390/cancers13112642/s1, Supplementary File S1: Hornheide Screening Instrument, Distress Thermometer, Supplementary File S2: first follow-up questionnaire, Supplementary File S3: second follow-up questionnaire.
