# Supplementary material for: Psychological Distress of Metastatic Melanoma Patients during Treatment with Immune Checkpoint Inhibitors: Results of a Prospective Study
Source: Cancers (Basel). 2021 May 27;13(11):2642. doi: 10.3390/cancers13112642 (PMC8198252; doi:10.3390/cancers13112642)
Supplement: Supplementary file 1 [file cancers-13-02642-s001.zip › Supplement 1.pdf]

Hornheide Screening Instrument

1. How did you feel physically in the last 3 days?	rather good<br>0	medium<br>1	rather poor<br>2
2. How did you feel mentally in the last 3 days?	rather good<br>0	medium<br>1	rather poor<br>2
3. Is there anything that weighs heavily on you regardless of the current illness?	yes<br>2	no<br>0	
4. Do you have someone to talk to about your fears and concerns?	yes<br>0	no<br>2	
5. Is anyone in your family particularly burdened by the hospital stay?	yes<br>2	no<br>0	
6. Can you find inner peace during the day?	yes<br>0	no<br>2	
7. How well do you feel informed about the disease and treatment?	rather good<br>1	medium<br>2	rather poor<br>3

Distress Thermometer

SCREENING TOOLS FOR MEASURING DISTRESS

Instructions: First please circle the number (0-10) that best describes how much distress you have been experiencing in the past week including today.

Extreme distress

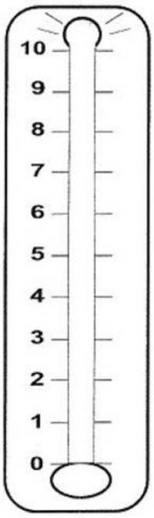

No distress

Second, please indicate if any of the following has been a problem for you in the past week including today. Be sure to check YES or NO for each.

YES NO **Practical Problems**
- ❑ ❑ Child care
- ❑ ❑ Housing
- ❑ ❑ Insurance/financial
- ❑ ❑ Transportation
- ❑ ❑ Work/school

Family Problems
- ❑ ❑ Dealing with children
- ❑ ❑ Dealing with partner

Emotional Problems
- ❑ ❑ Depression
- ❑ ❑ Fears
- ❑ ❑ Nervousness
- ❑ ❑ Sadness
- ❑ ❑ Worry
- ❑ ❑ Loss of interest in usual activities

❑ ❑ **Spiritual/religious concerns**

YES NO **Physical Problems**
- ❑ ❑ Appearance
- ❑ ❑ Bathing/dressing
- ❑ ❑ Breathing
- ❑ ❑ Changes in urination
- ❑ ❑ Constipation
- ❑ ❑ Diarrhea
- ❑ ❑ Eating
- ❑ ❑ Fatigue
- ❑ ❑ Feeling Swollen
- ❑ ❑ Fevers
- ❑ ❑ Getting around
- ❑ ❑ Indigestion
- ❑ ❑ Memory/concentration
- ❑ ❑ Mouth sores
- ❑ ❑ Nausea
- ❑ ❑ Nose dry/congested
- ❑ ❑ Pain
- ❑ ❑ Sexual
- ❑ ❑ Skin dry/itchy
- ❑ ❑ Sleep
- ❑ ❑ Tingling in hands/feet

Other Problems: ______________________________

____
