# Supplementary material for: Psychological Distress of Metastatic Melanoma Patients during Treatment with Immune Checkpoint Inhibitors: Results of a Prospective Study
Source: Cancers (Basel). 2021 May 27;13(11):2642. doi: 10.3390/cancers13112642 (PMC8198252; doi:10.3390/cancers13112642)
Supplement: Supplementary file 1 [file cancers-13-02642-s001.zip › Supplement 2.pdf]

Code:

University Hospital Tuebingen

University- Dermatology clinic
Medical Director: Prof. Dr. M. Röcken

Dr. A. Forschner

Tuebingen 07071/
29-83751 Operator
29-83768 Secretariat
29-84599 Melanoma consultation
29-4599 Fax

andrea.forschner@med.uni-tuebingen.de

First follow-up questionnaire

Dear Patient,

thank you again for agreeing to participate in our study. A few weeks have now passed since the start of therapy and you will receive the first follow-up questionnaire as discussed.

We kindly ask you to answer the following questions completely and then to fill out the enclosed distress thermometer, which you are already familiar with.
.

Date (please fill in): ___________________________

1. Have you had any personal contact with since starting current system therapy:
   - o a member of the psycho-oncology service here at the hospital?
   - o a psychooncologist/psychotherapist in private practice?
   - o other advice/support:____________________

2. Was the current system therapy able to be carried out as planned in your case up to now?
   - o yes
   - o no*

   *Why not?

   - o Side effects
   - o Lack of response of therapy
   - o Others:_____________________________

3. Have you experienced any side effects related to the therapy?
   - yes**
   - no

   ** If "Yes": how much do these side effects currently affect you (in the last week including today)?
     - rather few
     - medium
     - rather strong

We thank you very much for answering the questions and wish you a successful course of therapy and all the best!
(Please return the documents to us in the enclosed envelope).

Lisa Wiens
PhD student

Dr. A. Forschner,
Dermatologist, Psychooncologist
Head of the melanoma outpatient clinic

Dr. Norbert Schäffeler
Psychologist
